# Complement C3 and incident hospitalization due to chronic kidney disease: a population-based cohort study

**DOI:** 10.1186/s12882-019-1248-7

**Published:** 2019-02-21

**Authors:** Xue Bao, Yan Borné, Iram Faqir Muhammad, Christina-Alexandra Schulz, Margaretha Persson, Marju Orho-Melander, Kaijun Niu, Anders Christensson, Gunnar Engström

**Affiliations:** 1 Nutritional Epidemiology Institute and School of Public Health, Tianjin Medical University, Tianjin, China; 2 Department of Clinical Sciences, Lund University, Malmö, CRC 60:13, Jan Waldenströms gata 35, S-20502 Malmö, Sweden

**Keywords:** Chronic kidney disease, Complement C3, Diabetes, Hypertension

## Abstract

**Background:**

Circulating C3 has been associated with diabetes and hypertension, which are the leading causes of chronic kidney disease (CKD). C3 activation is considered to contribute to several renal diseases. Here we examined whether elevated C3 concentration is associated with hospitalization due to CKD in the general population, and whether this relationship is mediated by factors such as diabetes and hypertension.

**Methods:**

Baseline plasma C3 was quantified in 4552 participants, without previous hospital admission due to CKD, from the Malmö Diet and Cancer cohort study. Incidence of first hospitalization due to CKD (main diagnosis) was investigated in relation to C3 levels using Cox proportional hazards regression models after a mean follow-up of 19.2 ± 4.16 years. Traditional risk factors of CKD including diabetes, blood pressure, C-reactive protein and baseline renal function were considered in adjustments and sensitivity analyses.

**Results:**

During the follow-up period, 94 subjects were admitted to hospital due to CKD. After multivariate adjustment, the hazard ratios (95% confidence interval) for hospitalization from CKD across quartiles of C3 were 1.00 (reference), 1.68 (0.69, 4.13), 2.71 (1.15, 6.39), and 3.16 (1.36, 7.34) (*p* for trend = 0.003). Results were generally consistent across different sensitivity analyses.

**Conclusions:**

These findings indicate that C3 is associated with incidence of first hospitalization due to CKD in the general population. The observed relationship cannot be entirely attributed to hyperglycemia and hypertension.

## Background

Chronic kidney disease (CKD) is a heterogeneous group of disorders affecting kidney structure and function [1]. Currently, around 8–16% of people worldwide are suffering from it [2]. Many factors contribute to the development and progression of CKD, among which diabetes and hypertension are the foremost attributable causes in all developed and various developing countries [2]. Regardless of cause, CKD is associated with a substantially higher risk of cardiovascular events, hospitalization and mortality, and the association increases with the severity of CKD [2–5].

The complement system plays a crucial role in both innate and adaptive immune responses. While its deficiency leads to susceptibility to infection [6], its aberrant activation can cause or exacerbate various diseases [7]. Complement C3 is a central hub in the activation of the complement cascade [8]. Besides its role in complement cascade and inflammation, several observational studies have indicated that elevated C3 in plasma is associated with the development of diabetes and hypertension [9–11], i.e., two major causes of CKD [2]. C3 can be produced in the kidney [12, 13] and contributes to the circulating pool of C3 [14]. Sufficient evidence has accumulated in clinical settings to support the involvement of complement activation in renal damage [15]. In many kidney diseases, altered circulating C3 levels, renal C3 deposits, and genetic mutations in C3 can be observed [15]. It is thus possible that something in the renal structure or function could make kidney vulnerable to complement-mediated injury, and may directly contribute to the link between C3 and CKD.

Therefore, we examined the relationship between C3 and incidence of first hospitalization due to CKD in the general adult population. We also sought to explore whether the observed relation between C3 and CKD hospitalization was mediated by factors like diabetes and hypertension.

## Methods

### Participants

**Fig. 1:**
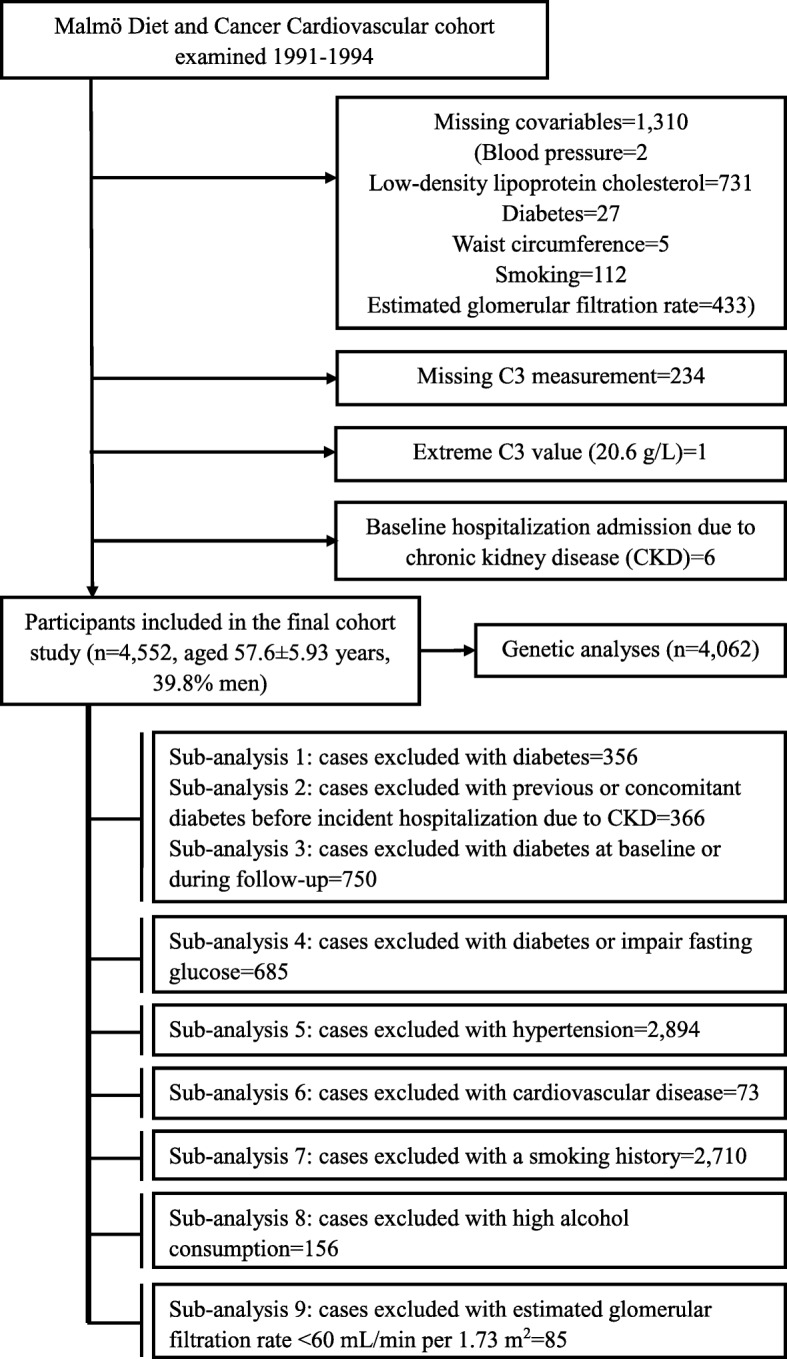
docx Study population flow chart

The Malmö Diet and Cancer study (MDCS) is a large prospective cohort study including both male and female residents from Malmö, a southern city of Sweden [16]. During 1991–1994, 6103 participants were randomly selected from the MDCS to study the epidemiology of carotid artery atherosclerosis [17]. They comprised the Malmö Diet and Cancer Cardiovascular (MDC-CV) cohort study. Among them, 4559 participants had complete information on C3 and confounders. We further excluded 1 participant with extremely high C3 value (20.6 g/L) and 6 participants with previous admission for CKD (Fig. 1). Therefore, the final population in the cohort analysis consisted of 4552 participants, with mean age of 57.6 ± 5.93 years, 39.8% of which were men. Written informed consent was obtained from all included participants. The study conformed to the Declaration of Helsinki and was approved by the ethical committee at Lund University, Lund, Sweden (LU 51/90).

### Baseline measurements and definitions

After an overnight fast, blood samples were drawn from the cubital vein and immediately stored at − 80 °C prior to testing. Fasting concentration of C3 (g/L), C-reactive protein (CRP, mg/L), creatinine (μmol/L) and cystatin C (mg/L) were all quantified from plasma samples. C3 was measured by an immunoturbidimetric method using Cobas c-systems and reagents from Roche Diagnostics, Germany. The assay shows a lower detection limit of 0.04 g/L and an upper limit of 5 g/L. Inter- and intra-coefficients of variation were 2.0 and 1.3%, respectively. LDL was calculated using Friedewald’s formula [18]. CRP was measured using the Tina-quant® CRP latex assay (Roche Diagnostics, Basel, Switzerland). Creatinine was analyzed in frozen plasma samples in 2009 with the Jaffé method, and traceable to the International Standardization with isotope dilution mass spectrometry. Cystatin C was measured using a particle-enhanced immunonephelometric assay (N Latex Cystatin; Dade Behring, Deerfield, IL) in 2009. Since measures were conducted before 2010 when the world calibrator was introduced, cystatin C values were not standardized (reference value: 0.53–0.95 mg/L) [19]. The estimated glomerular filtration rate (eGFR) was calculated based on the previously reported CKD- Epidemiology Collaboration 2012 equation [20] which takes both creatinine and cystatin C into consideration.

Systolic and diastolic blood pressures (BP) were measured in the left arm using a mercury-column sphygmomanometer after 10 min of rest in supine position. Waist circumference was measured midway between the lowest rib margin and iliac crest. Fasting blood glucose was measured according to standard procedures at the Department of Clinical Chemistry, University Hospital Malmö. Diabetes was defined as self-reported physician diagnosis of diabetes, use of anti-diabetic drugs or fasting whole blood glucose ≥6.1 mmol/L (corresponding to plasma glucose ≥7.0 mmol/L). A coronary event was defined based on the International Classification of Diseases 9th (ICD-9) codes 410A-410X and ICD-10 code I21 or death attributable to ischemic heart disease (ICD-9 codes: 410–414; ICD-10 codes: I20-I25). Stroke was defined as ICD-9 codes 430, 431,434 or 436, or ICD-10 codes I60, I61 or I63–64. Information regarding smoking habits, alcohol consumption and medication was collected in a questionnaire survey. Participants were categorized into current smokers, former smokers and never smokers. High alcohol consumption was defined as consuming an average of > 40 g pure alcohol per day for males, and > 30 g per day for females. Participants also reported whether they had been treated for inflammatory diseases, i.e., asthma/chronic bronchitis, rheumatoid arthritis, inflammatory bowel disease.

### Ascertainment for incidence hospitalization due to CKD

In the present study, information on hospitalization due to CKD was obtained from the Swedish patient register. This register covers all hospitalizations in south of Sweden since 1970 and has nation-wide coverage since 1987. From 2001 onwards, all out-patient hospital visits are also included in the register. The register has been previously described and validated for outcome classification [21]. In addition, the Swedish renal registry was searched for any additional cases of CKD [22]. CKD was defined as codes 585–586 according to ICD-9, and N18 and N19 according to ICD-10. Hospitalization due to CKD was defined as first admission to hospital for having CKD as the main diagnosis. In a sensitivity analysis, we also examined the relationship between C3 and incidence of hospitalization due to CKD, with CKD listed as the main or contributing diagnosis (one of the first three positions). In 100 randomly selected patients from MDCS, the CKD diagnoses were evaluated by two experienced specialists in nephrology using patient records and laboratory data. A CKD diagnosis requires two measuring points at least 3 months apart to meet the criteria (KDIGO, CKD Work Group 2012 [23]). The diagnoses were divided into 4 groups based on the degree of reliability: grade 0, incorrect diagnosis; grade 1, low probability of correct diagnosis, or insufficient information (e.g. GFR 58 ml/min at one time only and no indication of albuminuria); grade 2, reasonable high probability of correct diagnosis (e.g. 2.5 months between creatinine analyzes instead of 3 months); grade 3, correct diagnosis. Grades 0–1 was considered incorrect. Grades 2–3 were considered to be correct. The final result showed 94 correct diagnoses, 5 incorrect diagnoses, and 1 case that cannot be classified as correct due to doubtful or insufficient data. Thus, the validation showed that 94% of the patients had a correct diagnosis of CKD.

All participants without any previous hospitalization admission due to CKD at baseline were followed from the date of the baseline examinations until the occurrence of a hospital diagnosis of CKD, emigration from Sweden, death or December 31st, 2013, whichever came first.

### Statistical analyses

For skewed variables, log-transformed values were used to obtain a normal distribution before analyses. Multiple linear regression (adjusted for age and sex) was used for continuous variables to compare differences in baseline characteristics between individuals with and without hospitalization due to CKD during follow-up, and multiple logistic regression analysis was used for categorical variables. Participants were then divided into quartiles based on C3 levels. The association between C3 quartiles and incidence of hospitalization due to CKD was estimated with Cox proportional hazard regression and expressed as hazard ratios (HR) with 95% confidence intervals (CIs). Timescale was time to follow-up until incident CKD hospitalization, emigration, death or end of follow-up. Potential confounders including age, sex, waist circumference, smoking and drinking habits, LDL cholesterol, diabetes, cardiovascular disease (CVD), use of anti-hypertensive drug medications, and baseline eGFR were all included in the final model.

Potential interactions between C3 and covariates were tested by adding interaction terms in the final model. In sensitivity analyses, the final model was repeated with baseline creatinine, cystatin C, hsCRP, and inflammatory diseases (i.e., asthma/chronic bronchitis, rheumatoid arthritis, inflammatory bowel disease) additionally taken into consideration. In addition, Fine and Grey models [24, 25] were used to determine the subdistribution HR for hospitalization due to CKD, and death was accounted for as a competing event. We also conducted various subgroup analyses using C3 as a continuous variable (Fig. 1), in which we respectively excluded participants with 1) baseline diabetes; 2) previous or concomitant diabetes before incident hospitalization for CKD; 3) baseline diabetes or incident diabetes during follow-up; 4) baseline diabetes or impaired fasting glucose (IFG, fasting glucose ≥5.6 mmol/L); 5) baseline hypertension; 6) baseline CVD; 7) history of smoking; 8) high alcohol consumption; 9) impaired renal function at baseline (eGFR< 60 mL/min per 1.73 m^2^).

The restricted cubic spline function was applied to detect possible non-linearity, with knots placed at the 5th, 25th, 50th, 75th, and 95th percentiles of C3 concentration. All analyses were performed using the Statistical Analysis System version 9.3 for Windows (SAS Institute Inc., Cary, NC, USA).

## Results

### Baseline characteristics

Age- and sex-adjusted baseline characteristics of participants with and without CKD hospitalization during follow-up are presented in Table 1. Participants who were hospitalized due to CKD during follow-up had significantly higher C3 levels at baseline. They also had lower eGFR, and higher fasting glucose, systolic BP, diastolic BP, waist circumference, and usage of anti-hypertensive medication.

**Table 1:** Baseline characteristics by status of incident hospitalization due to chronic kidney disease (CKD) during follow-up

	Incidence hospitalization due to CKD		*p* ^a^
	No (*n* = 4458)	Yes (*n* = 94)	
Age (years)	57.3 (57.1, 1.93) ^b^	59.1 (57.8, 3.14)	0.004
Sex (male, %)	39.5	56.4	<0.001
Complement C3 (g/L)	1.51 (1.50, 1.52)	1.71 (1.64, 1.78)	<0.001
Fasting glucose (mmol/L)	5.06 (5.04, 5.09)	5.71 (5.51, 5.92)	<0.001
Systolic blood pressure (mmHg)	140.2 (139.6, 8.64)	146.4 (142.8, 9.65)	<0.001
Diastolic blood pressure (mmHg)	86.6 (86.3, 13.6)	89.5 (87.6, 15.5)	<0.001
Waist circumference (cm)	83.9 (83.6, 7.58)	90.1 (88.0, 10.2)	<0.001
Low-density lipoprotein cholesterol (mmol/L)	4.04 (4.01, 10.7)	4.10 (3.91, 7.46)	0.54
eGFR (mL/min per 1.73 m^2^)	88.8 (88.4, 8.27)	73.0 (70.9, 8.13)	<0.001
Cystatin C (mg/L)	0.76 (0.76, 5.07)	0.93 (0.90, 8.36)	<0.001
Creatinine (μmol/L)	84.3 (84.0, 14.3)	95.5 (92.6, 4.53)	<0.001
High alcohol consumption (%)	3.45	3.19	0.76
Smokers (%)	21.6	24.5	0.40
Anti-hypertensive medication (%)	16.1	38.3	<0.001
Diabetes (%)	7.51	22.3	<0.001
Cardiovascular disease (%)	2.15	7.45	0.02

eGFR, estimated glomerular filtration rate according to the 2012 Chronic Kidney Disease Epidemiology Collaboration equation

^a^Multiple linear or logistic regression analysis, adjusted for age and sex

^b^Geometric mean (95% confidence interval) (all such values)

The distribution of risk factors across C3 quartiles is shown in Table 2. Compared to participants in the lowest quartile of C3, those in the higher C3 quartile had significantly lower baseline eGFR. Meanwhile, they were more likely to be older, to be male or non-smokers, and to have higher fasting glucose, systolic BP, diastolic BP, waist circumference, LDL, cystatin C, and usage of anti-hypertensive medication, and to suffer from diabetes or CVD.

**Table 2:** Age and sex-adjusted characteristics of individuals across complement C3 quartiles (Q1-Q4)

	C3 quartiles				*P* for trend ^a^
	Q1	Q2	Q3	Q4	
No. of subjects	1069	1195	1145	1143	–
Age (years)	56.2 (55.9, 56.6) ^b^	57.3 (56.9, 57.6)	57.9 (57.5, 58.2)	57.7 (57.4, 58.1)	<0.001
Sex (male, %)	34.4	41.7	42.5	40.2	<0.001
Fasting glucose (mmol/L)	4.85 (4.80, 4.90)	4.95 (4.90, 5.00)	5.12 (5.07, 5.17)	5.38 (5.33, 5.44)	<0.001
Systolic blood pressure (mmHg)	136.6 (135.6, 137.6)	138.6 (137.7, 139.6)	142.4 (141.4, 143.5)	143.4 (142.4, 144.4)	<0.001
Diastolic blood pressure (mmHg)	84.3 (83.8, 84.8)	85.9 (85.4, 86.4)	87.4 (86.9, 87.9)	88.8 (88.3, 89.3)	<0.001
Waist circumference (cm)	78.6 (78.0, 79.1)	82.2 (81.7, 82.7)	85.5 (84.9, 86.0)	90.1 (89.5, 90.7)	<0.001
Low-density lipoprotein cholesterol (mmol/L)	3.77 (3.72, 3.82)	4.00 (3.95, 4.06)	4.13 (4.07, 4.19)	4.24 (4.18, 4.30)	<0.001
eGFR (mL/min per 1.73 m^2^)	89.0 (88.2, 89.8)	88.1 (87.4, 88.9)	88.8 (88.1, 89.6)	87.6 (86.9, 88.4)	0.04
Cystatin C (mg/L)	0.75 (0.75, 0.76)	0.77 (0.76, 0.77)	0.77 (0.76, 0.77)	0.78 (0.77, 0.79)	<0.001
Creatinine (μmol/L)	84.9 (84.1, 85.7)	85 (84.2, 85.7)	84.1 (83.3, 84.8)	84.4 (83.6, 85.2)	0.11
High alcohol consumption (%)	3.37	3.26	3.84	3.24	0.99
Smokers (%)	23.9	23.5	19.0	20.3	<0.001
Anti-hypertensive medication (%)	10.3	13.4	17.2	24.9	<0.001
Diabetes (%)	2.90	4.35	8.03	15.8	<0.001
Cardiovascular disease (%)	1.59	1.67	1.83	3.94	<0.001

eGFR, estimated glomerular filtration rate according to the 2012 Chronic Kidney Disease Epidemiology Collaboration equation

^a^Multiple linear or logistic regression analysis, adjusted for age and sex

^b^Geometric mean (95% confidence interval) (all such values)

### Incidence of hospitalization due to CKD in relation to C3

During a mean follow-up of 19.2 ± 4.16 years, 94 participants (2.07%, 53 men and 41 women) were hospitalized due to CKD (main diagnosis). Incident hospitalization due to CKD was 1.08 per 1000 person-years. Fifty-four cases developed CKD stage 4 or more, and 21 cases progressed to end stage renal disease (ESRD) before end of follow-up.

As shown in Table 3, an increase in baseline C3 was associated with a substantially increased risk of CKD hospitalization. In the crude model, the HR and 95% CI (highest vs. lowest quartiles of C3) for CKD hospitalization was 6.46 (2.91–14.3; *p* for trend< 0.001). When adjusted for age and sex, this value changed marginally (HR, 5.82; 95% CI: 2.62–12.9; *p* for trend< 0.001). Further adjustment for other covariates attenuated the HR to 3.16 (95% CI: 1.36–7.34; *p* for trend = 0.003). If fasting glucose was adjusted for instead of diabetes, the risk increase was rather similar (HR for the highest vs. lowest quartiles, 2.96; 95% CI, 1.28–6.86; *p* for trend = 0.004; data not shown). In 4443 participants with available data on hsCRP, we additionally adjusted for CRP, but the risk persisted (2.60, 1.09–6.20; *p* for trend = 0.022; data not shown). Furthermore, of the 4527 participants with information available, 518 reported that they had inflammatory diseases (asthma/chronic bronchitis, rheumatoid arthritis or inflammatory bowel disease). Results were substantially unchanged when this confounder was additionally adjusted for in multivariate analyses (HR for the highest vs. lowest quartiles, 3.17; 95% CI, 1.36–7.38; *p* for trend = 0.003; data not shown). No significant interaction was observed between C3 and other covariates. The restricted cubic spline analysis did not show any evidence for non-linearity (*p* non-linearity = 0.26). The age- and sex-adjusted HR (Q4 vs Q1) for those who developed CKD stage 4 or more before end of follow-up was 1.49 (95% CI, 1.15–1.94; *p* for trend = 0.003). The age- and sex-adjusted HR was not significant for the 21 cases that developed ESRD (HR, 1.11; 95% CI, 0.75–1.66; *p* for trend = 0.59).

**Table 3:** Incidence of hospitalization due to chronic kidney disease in relation to complement C3 quartiles (Q1-Q4)

	Quartile 1	Quartile 2	Quartile 3	Quartile 4	*P* for trend ^a^
C3 range (g/L)	<1.3	1.31–1.49	1.50–1.71	>4.05	
No. of subjects	1069	1195	1145	1143	–
Incidence (main diagnosis) ^b^	7	16	26	45	–
Incidence (per 1000 person-years)	0.33	0.69	1.20	2.09	–
Model 1 ^d^	Reference	2.09 (0.86, 5.07) ^c^	3.63 (1.58, 8.36)	6.46 (2.91, 14.3)	<0.001
Model 2 ^e^	Reference	1.86 (0.77, 4.53)	3.14 (1.36, 7.24)	5.82 (2.62, 12.9)	<0.001
Model 3 ^f^	Reference	1.68 (0.69, 4.13)	2.71 (1.15, 6.39)	3.16 (1.36, 7.34)	0.003
Incidence (main or contributing diagnosis) ^g^	20	38	51	77	–
Incidence (per 1000 person-years)	0.96	1.65	2.35	3.60	–
Model 1 ^d^	Reference	1.74 (1.01, 2.98)	2.50 (1.49, 4.19)	3.89 (2.38, 6.37)	<0.001
Model 2 ^e^	Reference	1.50 (0.87, 2.58)	2.07 (1.23, 3.48)	3.46 (2.12, 5.66)	<0.001
Model 3 ^f^	Reference	1.39 (0.81, 2.40)	1.50 (0.88, 2.55)	1.73 (1.02, 2.92)	0.047

^a^Analysis by Cox proportional hazards model

^b^Incidence of hospitalization due to chronic kidney disease (defined as 585–586 according to International Classification of Diseases (ICD) 9, and N18 and N19 according to ICD10; main diagnosis refer to cases at the first position; main or contributing diagnosis refer to cases at one of the first three positions)

^c^Adjusted hazard ratios (95% confidence interval) (all such values)

^d^Crude model

^e^Adjusted for age and sex

^f^Adjusted for age, sex, waist circumference, smoking and drinking habits, low-density lipoprotein cholesterol, diabetes, cardiovascular disease, anti-hypertensive drug medication, and baseline estimated glomerular filtration rate

During the follow-up period, 480 participants died without CKD hospitalization. Using death as a competing event, the overall findings were relatively unchanged. The subdistribution HR (95% CI) for CKD hospitalization was 3.14 (1.36–7.23; *p* for trend = 0.001) in the highest vs lowest quartile of C3 and was 1.33 (1.14–1.55; *p* < 0.001) per 1 standard deviation (SD) increase in C3.

When C3 was used as a continuous variable, the multivariate-adjusted HR of CKD hospitalization increased by 28% for each 1 SD increment in C3 concentration. In subgroup analyses, the association between C3 and CKD hospitalization remained significant in participants without baseline diabetes (1.35, 1.10–1.66, *p* = 0.004), without previous or concomitant diabetes before incident hospitalization for CKD (1.39, 1.11–1.73, *p* = 0.004), without baseline diabetes or incident diabetes during follow-up (1.46, 1.17–1.82, *p* = 0.001) and in those with normal fasting glucose at baseline (1.43, 1.13–1.81, *p* = 0.003). Similarly, as shown in Table 4, the association was significant in participants without hypertension and in other subgroups.

**Table 4:** Adjusted HR (95% CI) for incident CKD hospitalization per 1 SD increase in complement C3 ^a^

	Mean ± SD of C3	No. of subjects	Incidence	HR (95%CI)	*P*
Whole study population	1.548 ± 0.353	4552	94	1.28 (1.08, 1.53)	0.005
Model 1 ^b^	1.529 ± 0.341	4196	73	1.35 (1.10, 1.66)	0.004
Model 2 ^c^	1.528 ± 0.341	4186	63	1.39 (1.11, 1.73)	0.004
Model 3 ^d^	1.511 ± 0.330	3802	63	1.46 (1.17, 1.82)	0.001
Model 4 ^e^	1.515 ± 0.332	3867	64	1.43 (1.13, 1.81)	0.003
Model 5 ^f^	1.475 ± 0.343	1658	81	1.65 (1.16, 2.35)	0.005
Model 6 ^g^	1.544 ± 0.351	4479	87	1.27 (1.06, 1.52)	0.010
Model 7 ^h^	1.548 ± 0.350	1842	32	1.57 (1.18, 2.09)	0.002
Model 8 ^i^	1.548 ± 0.354	4396	91	1.26 (1.06, 1.51)	0.011
Model 9 ^j^	1.546 ± 0.352	4467	79	1.25 (1.04, 1.50)	0.018

^a^*HR* hazard ratios, *CI* confidence interval, *CKD* chronic kidney disease, *SD* standard deviation; adjusted for age, sex, waist circumference, smoking and drinking habits, low-density lipoprotein cholesterol, diabetes, cardiovascular disease, anti-hypertensive drug medication, and baseline estimated glomerular filtration rate (specific covariate was not adjusted when being investigated in a specific model)

^b^Excluding participants with baseline diabetes

^c^Excluding participants with previous or concomitant diabetes before incident chronic kidney disease

^d^Excluding participants with baseline diabetes or incident diabetes during follow-up

^e^Excluding participants with baseline diabetes or impair fasting glucose (fasting glucose ≥5.6 mmol/L)

^f^Excluding participants with baseline hypertension

^g^Excluding participants with baseline cardiovascular disease

^h^Excluding current, occasional or former smokers

^i^Excluding participants with high alcohol consumption

^j^Excluding participants with estimated glomerular filtration rate < 60 mL/min per 1.73 m^2^

A total of 186 individuals were hospitalized due to CKD as the main or contributing diagnosis (one of the first three positions). We also explored the relationships between C3 and CKD hospitalization using this endpoint definition. After adjustments in the final model, the HR for hospitalization due to CKD was 1.73 (1.02, 2.92; *p* for trend = 0.047) comparing the 4th vs 1st quartile (Table 3).

## Discussion

The present population-based cohort study showed that elevated plasma C3 was associated with increased incidence of first hospitalization due to CKD among middle-aged adults. This relationship was independent of traditional risk factors including diabetes, blood pressure, baseline renal function and CRP.

C3 is an acute phase protein and a protein crucial to all complement activation pathways [8]. Hence, raised C3 levels could be a result of systemic low-grade inflammation, while low levels are observed in conditions characterized by excessive complement activation [15]. C3 is richly expressed in abdominal adipose tissue as well as kidneys [13, 14, 26]. Plasma lipids have been reported to be associated with the development of CKD [27], and the coexistence of hypertriglyceridemia and high C3 levels showed a strong association with proteinuria [28]. In addition, both obesity and elevated C3 levels were related to higher risk of insulin resistance and diabetes [9, 29], a primary cause of CKD [2]. These findings indicate that dysregulation of the lipid and glucose metabolism could be a common link between raised C3 and CKD. In accordance with this view, we found that C3 was positively associated with LDL, waist circumference, fasting glucose and diabetes at baseline. However, the longitudinal relationship between C3 and CKD hospitalization remained significant after extensive exclusions of those with diabetes or impaired fasting glucose. Similarly, whereas hypertension may potentially confound our primary finding [10, 30], the relationship between C3 and CKD hospitalization remained significant in non-hypertensive subjects. Thus, it is unlikely that the relation between C3 and CKD hospitalization was entirely mediated by diabetes or hypertension. In addition, adjusting for baseline eGFR or excluding participants with eGFR< 60 mL/min per 1.73 m^2^ only marginally influenced the impact of C3 on risk for CKD hospitalization. Other sensitivity analyses, adjustments and tests of interaction consistently supported an independent predictive value of C3 for incident hospitalization for CKD. This conclusion was further confirmed by results obtained from analyses accounting for the competing risk of death.

Inflammation has been shown to be involved in the development of kidney disease in both experimental and epidemiological studies [31, 32]. However, the results changed only marginally when inflammatory diseases were taken into consideration in multivariate adjustments. In addition, the association of C3 with hospitalization due to CKD decreased but remain significant even after adjusting for CRP, another common inflammatory marker. This is in line with previous observations suggesting that there was no significant association between CRP levels and incident CKD hospitalization [31], and that C3 may be preferable to CRP as an inflammatory marker of cardiometabolic traits [11, 33, 34].

Noteworthily, the kidney itself is an important source of extrahepatic C3 [12, 13], and contributes significantly to circulating C3 [14]. C3 gene expression has been widely detected in normal human renal tissue and can be substantially increased by multiple stimuli [13]. In podocytes, which are critical for the integrity of the glomerular filtration barrier, the C3 synthesis is up-regulated in response to injury or cellular stress [12]. Local C3 production correlates closely with the severity of kidney tissue injury, probably via enhanced T- and B-cell function, recruitment of pro-inflammatory and pro-fibrotic cytokines, disposal of immune complexes and apoptotic cells, *etc* [13]. Plasma C3, as partially originated from the kidney, could be a marker of ongoing injury of kidney cells, which ultimately results in CKD. Whereas no previous study has explored the association between circulating C3 and CKD in the general population, higher serum C3 levels have been found to be associated with higher levels of urine protein in patients with non-nephrotic CKD [28], and with renal arteriolosclerosis in patients with non-diabetic CKD [35]. Decreased blood C3 levels have been observed in some less common renal diseases (e.g. atypical hemolytic uremic syndrome, dense deposit diseases, and C3 glomerulonephritis [15, 36, 37]), perhaps due to aberrant complement activation or impaired glomerular filtration barrier and proteinuria [38]. However, these nephropathies constitute a minor proportion of all CKD cases. In contrast, the present study was conducted in the general population, and we demonstrated that elevated C3 levels were associated with decreased eGFR at baseline and CKD hospitalization during follow-up. Since the kidney generally contributes to human circulating C3 concentration in a significant and sustained manner [14], it is speculated that high plasma C3 in apparently healthy individuals could reflect a mild and subclinical complement activation that ultimately results in increased risk of CKD hospitalization.

### Strengths and limitation

This study had a prospective study design with long follow-up duration. The endpoint was retrieved from hospital registers with national coverage. The CKD diagnosis in this study was validated with a correct rate of 94%. Moreover, a variety of sensitivity analyses were conducted, and the results remained significant. However, due to the descriptive nature of the observational design, our findings provide limited insight into the underlying mechanism. Many patients were treated in primary care and it was obvious that some individuals with less severe renal dysfunction were missed. The CKD cases in this study constituted of cases who were treated in hospital, which assumingly were the most severe cases. The use of ICD codes to identify the cause of hospital admission might be sub-optimal to CKD based on eGFR measurements. Our study is also limited by a low incidence of CKD hospitalization, which may reduce the statistical power. Due to the observational design of the study, findings are descriptive in nature. Moreover, the cohort only included participants being residents of Malmö, Sweden, further studies are needed to generalize the results to other populations.

## Conclusions

Our results revealed that C3 is associated with incidence of CKD hospitalizations in the general population, independent of traditional risk factors including diabetes, blood pressure, CRP and baseline renal function.

## Abbreviations

*BP*: blood pressure
*CI*: confidence interval
*CKD*: Chronic kidney disease
*CRP*: C-reactive protein
*CVD*: cardiovascular disease
*eGFR*: estimated glomerular filtration rate
*HR*: hazard ratio
*ICD*: International Classification of Disease
*IFG*: impaired fasting glucose
*LDL*: low-density lipoprotein cholesterol
*MDC-CV*: Malmö Diet and Cancer Cardiovascular
*MDCS*: Malmö Diet and Cancer study
*SD*: standard deviation
